# Examining the accuracy of students’ self-reported academic grades from a correlational and a discrepancy perspective: Evidence from a longitudinal study

**DOI:** 10.1371/journal.pone.0187367

**Published:** 2017-11-07

**Authors:** Fabio Sticca, Thomas Goetz, Madeleine Bieg, Nathan C. Hall, Franz Eberle, Ludwig Haag

**Affiliations:** 1 Department of Empirical Educational Research, University of Konstanz, Konstanz, Germany & Thurgau University of Teacher Education, Kreuzlingen, Switzerland; 2 Department of Empirical Educational Research, University of Konstanz, Konstanz, Germany; 3 Department of Educational and Counselling Psychology, McGill University, Montreal, Canada; 4 Institute of Education, University of Zurich, Zurich, Switzerland; 5 Department of Education, University of Bayreuth, Bayreuth, Germany; Technische Universitat Chemnitz, GERMANY

## Abstract

The present longitudinal study examined the reliability of self-reported academic grades across three phases in four subject domains for a sample of 916 high-school students. Self-reported grades were found to be highly positively correlated with actual grades in all academic subjects and across grades 9 to 11 underscoring the reliability of self-reported grades as an achievement indicator. Reliability of self-reported grades was found to differ across subject areas (e.g., mathematics self-reports more reliable than language studies), with a slight yet consistent tendency to over-report achievement levels also observed across grade levels and academic subjects. Overall, the absolute value of over- and underreporting was low and these patterns were not found to differ between mathematics and verbal subjects. In sum, study findings demonstrate the consistent predictive utility of students’ self-reported achievement across grade levels and subject areas with the observed tendency to over-report academic grades and slight differences between domains nonetheless warranting consideration in future education research.

## Introduction

Academic grades are the most common indicators of academic achievement, one of the most investigated constructs in educational research [1], and are consistently associated with a range of academic variables including self-concept [2] and emotions [3], as well as with non-academic ones such as socioeconomic status [4]. In many studies, self-reported academic grades are evaluated as proxies of actual performance based largely on the pragmatic rationale that self-reported grades are considerably more accessible and efficient. However, although the use of self-reports is widely accepted in the social sciences [5,6], these measures are unfortunately susceptible to systematic and random error that can contribute to biased results.

Given their widespread use, it is important to specifically examine the accuracy of self-reported grades to ascertain the extent of potential errors in estimating achievement levels. To date, most research on the accuracy of self-reported grades has been conducted in school systems in which the use of grade point averages (GPA) is common (i.e., North America). Accordingly, considerably less is known as to the accuracy of self-reported achievement in countries with different grading practices (e.g., European 6-point grading scale). We additionally lack knowledge on the extent to which the accuracy of self-reported grades differs across grade levels and academic subjects (e.g., mathematics vs. verbal domains). The present study thus aimed to address these research gaps by examining the accuracy of students’ self-reported grades in an underexplored educational context (Switzerland) and further accounting for potential differences across age levels (grades 9–11) and academic subjects (mathematics vs. language classes).

### Indicators of the accuracy of self-reported grades

When examining the accuracy of a given self-report measure, it is crucial to first operationalize the specific manner in which the accuracy is to be assessed. In the context of the present study, the accuracy of self-reported grades was examined as indicative of actual grades and *not* as indicative of academic ability. The accuracy of self-reported grades (note that in the following, the expression accuracy of self-reported grades will be used to indicate accuracy of self-reported grades as indicators of actual grades) can be illustrated by correlations between self-reported and actual grades as well as differences between the levels of each (i.e., difference scores). From the correlational perspective, the focus lies on the extent to which self-reported and actual grades correlate. According to classical test theory [7], the correlation between self-reported grades and actual grades represents an estimation of the reliability of self-reported grades. For a detailed discussion about the reliability of self-reported grades, see Kuncel, Credé, and Thomas [1].

As such, if the research question of a given study is simply to examine correlations between academic grades and other academic variables, a high degree of reliability concerning self-reported grades might be sufficient to justify their use. However, for other research questions, such as those concerning how self-reported grades may systematically differ from actual grades for different groups (e.g., gender, grade levels), demonstrating the reliability of self-reported grades is not sufficient as high correlations can be found despite systemic under- or over-reporting of actual grades.

When computing the difference between self-reported and actual grades (i.e., self-reported minus actual grades: *sg-ag*), the resulting difference score conveys the accuracy of a student’s self-reported grade, which will be indicated as *Diff*_*_*_*sg-ag* in the following. For this measure, a positive value reflects *over-reporting*, zero reflects an accurate report, and a negative value reflects *under-reporting*. The simple mean of the difference scores across all subjects may also be assessed and will be labeled *mean misreporting* and indicated with *M*_Diff_sg-ag_ in the following. Mean misreporting indicates the overall direction and extent of imbalance between over- and under-reporting of class grades more generally (i.e., a positive value would indicate more over- than under-reporting, on average). Finally, the mean of the *absolute* values of the differences can be calculated as a mean across all absolute differences *|Diff*_*_*_*sg-ag|* of all students and will be labeled *absolute misreporting* and indicated with *M*_|Diff_sg-ag|_ in the following. The scores of absolute misreporting can be used to examine the extent of incorrect reporting without differentiating between over- and under-reporting. In sum, the discrepancies perspective allows for differences between self-reported and actual grades to be specifically assessed so as to more closely examine the overall extent and directions of misreporting and thus contribute a more comprehensive picture of the accuracy of self-reported grades than correlations alone.

### State of research on the accuracy of self-reported grades

The accuracy of self-reported grades has been examined in several studies conducted primarily with North American samples. Kuncel et al. [1] conducted a large-scale meta-analysis of studies evaluating the accuracy of students’ self-reported GPAs, class ranks, and SAT scores published prior to 2003 resulting in 37 independent samples encompassing 60,926 individuals. The average correlation between self-reported and actual grades was .84, with correlations of .90 observed for college students (*N* = 12,089) and .82 for high-school students (*N* = 44,176). Their results also showed that self-reported grades were more reliable indicators of actual grades for students with higher levels of academic performance and cognitive ability. Further, demographic variables were not found to moderate the reliability of self-reported grades, except for minority student status, which corresponded with lower reliability. Finally, school subject significantly moderated the reliability of self-reported GPAs, with the lowest reliability found for arts and music and the highest for social science. From a discrepancies perspective, students tended to over-report their grades with the percentage of students over-estimating their grades being three to four times higher than the proportion of students who under-reported their performance.

Of the few studies on the accuracy of self-reported grades conducted subsequent to the meta-analysis by Kuncel et al. [1], most have primarily replicated the results of the meta-analysis. From a correlational perspective, all studies have found self-reported grades to be reliable indicators of actual grades [8–16]. However, some studies showed moderation effects, such as Schneider and Sparfeldt [10] who found self-reported grades to be more reliable indicators for fourth-graders than third- and second-grade students. Concerning other potential moderating factors, the reliability of self-reported grades was not found to significantly differ as a function of gender [8,12,13,16] with the exception of findings from Feng and Rost [14] who found self-reported grades to be slightly higher for females with respect to mathematics, History, and Chinese (first language). Existing research has also shown academic discipline to not significantly moderate the reliability of self-reported grades [13].

Concerning discrepancies between self-reported and actual grade levels, studies have consistently shown a slight tendency for students to over-report their grades [8–11,13–18]. However, one exception in this regard is a study by Shaw and Mattern [12] who found a slight tendency for high-school students to under-report their grades. There to date remain no published studies examining the extent of absolute misreporting irrespective of over/under-reporting.

Notably few studies on the accuracy of self-reported grades having been conducted in German-speaking countries [8–10,18,19] and, to the best of our knowledge, no published studies to date have explored the accuracy of self-reported grades within the Swiss school system. In existing studies in such settings, the accuracy of self-reported grades was examined mainly in mathematics, with the two studies evaluating other subjects (e.g., German, English, and sports) showing no major differences in the reliability of self-reported grades [10,17,18]. As such, there exists a specific need to further examine not only the accuracy of students’ self-reported grade in European education settings, but also to more extensively examine how these relations may differ by subject area.

Further study within the Swiss grading system as an example of a European education setting is also warranted for two additional reasons. First, a measure comparable to cumulative GPA is not commonly employed in many European education settings, with greater emphasis instead being given to grades in specific academic subjects as opposed to across subjects. Second, there are notable differences in grading practices in European countries that limit the comparability of grades. Whereas GPA values typically range from 0 to 4 (i.e., E/F, D, C, B, and A with further differentiation through the use of +/-), Swiss grades range from 1 (poorest grade) to 6 (best grade) with 4 being the threshold for passing and further differentiation through the use of half grades (e.g., 4.5; cf. Germany: 6 = poorest, 1 = best; Italy: 1 = poorest, 30 = best). As such, this greater degree of differentiation in student grading could result in different likelihoods of misreporting than those observed in studies based on North American samples. In sum, knowledge as to the accuracy of self-reported grades in Swiss education system is lacking both generally and within specific subject domains, despite self-reported grades being commonly used as indicators of actual grades in Swiss educational research [20,21].

### Aims and hypotheses

The present longitudinal study examined the accuracy of self-reported grades in four academic subjects in a sample of Swiss high-school students (grades 9 to 11) from both a *correlational* and a *discrepancies perspective*. Following from meta-analytic findings by Kuncel et al. [1], we hypothesized that, from a correlational perspective, self-reported and actual grades would be positively correlated in all academic subjects at all measurement occasions. As for differences in the reliabilities across domains, no specific hypotheses could be derived from previous research. Given previous results on the reliability of self-reported grades in mathematics, we aimed to specifically compare both correlations and discrepancy levels for the mathematics domain as well as multiple verbal domains (i.e., German, English, and French). Regarding the discrepancies perspective, we expected to find positive mean misreporting as is consistently observed in previous research [1]. With respect to absolute misreporting and self-report/actual grade discrepancies across subjects, no specific hypotheses were once again able to be derived from previous research.

## Method

### Ethics statement

The present study was conducted in compliance with ethical standards expressed in the Swiss Federal Act on Research on Human Beings, the WMA Declaration of Helsinki, the Federation of German Psychologists Association, and the American Psychological Association. Furthermore, the study has been approved and all study procedures have been deemed appropriate by the Institutional Review Board of the University of Konstanz. Guidelines provided by these institutions state that formal informed consent is not obligatory when no potential harm or distress is to be expected and/or when normal educational practices are followed as a goal of the research. Nevertheless, prior to each assessment, participants were informed that participation in the study was voluntary and that they could discontinue their involvement at any time. All parents and caregivers were informed of the study aims and procedures, with all protocols approved by schools principals and teachers. A copy of the entire questionnaire as well as a list encompassing all data that were to be collected, including actual academic grades, was outlaid in the secretariat of all participating schools and all participants and their parents had the possibility to review it. Furthermore, during assessments some students mentioned that they did not remember their exact grade and were informed that they could simply report the grade that they remembered and that actual grades would also be reported by the secretariat. All identifiers that could link individual participants to their results were removed and destroyed after data entry. Hence, all analyses were conducted on depersonalized data. After each assessment, participants were compensated with a small gift, such as chocolate, and were entered into a prize draw (iPod).

### Sample and procedure

The present longitudinal study was conducted with high-school students recruited across multiple German-speaking Swiss cantons. Assessments began in the spring of 2012 (T1: grade 9), continued in the spring 2013 (T2: grade 10), and terminated in the spring of 2014 (T3: grade 11). Of all possible German-speaking upper-track schools available for recruitment in which mathematics, German (local language), English, and French were taught, eight schools were selected for participation with students recruited from 45 grade 9 classrooms. Participants totals included 916 students at T1 (56.1% female; mean age 15.6 years, *SD* = .63), 719 at T2 (55.5% female; mean age 16.6 years, *SD* = .63), and 647 at T3 (55.3% female; mean age 17.7 years, *SD* = .75). A total of 571 (57.4%) students participated in all three assessments. Attrition was due primarily to one school withdrawing from the study after the first assessment due to organizational reasons that were in no way related to the study itself, to students relocating to another school, or to students being absent during data collection. Assessments were carried out in the classrooms during a single lesson using a paper and pencil questionnaire.

### Study measures

#### Demographic variable

Participants’ gender and age were obtained via self-report.

#### Actual grades

Each student’s midyear grades (i.e., obtained each December, four months prior to a given assessment) in mathematics, German, English, and French were provided by school administrators at each assessment. Table 1 shows the mean scores and standard deviations of actual grades.

**Table 1:** Descriptive statistics, correlations, T-Test for the difference between self-reported and actual grades, and means of misreporting.

	*Descriptive Statistics*					*Paired Sample t-Tests for M*_*Diff_sg-ag*_					*Means of Misreporting*		
	*M*_*sg*_	*SD*_*sg*_	*M*_*ag*_	*SD*_*ag*_	*r*_*sg-ag*_	*M*_*Diff_sg-ag*_	*SD*	*SE*	*t*	*d*	*M*_|Diff_sg-ag|_	*M*_*Over*_	*M*_*Under*_
T1 (Grade 9)													
Mathematics	4.57	0.74	4.52	0.75	.93***	0.046***	0.28	0.01	4.83	0.16	0.107	0.077	-0.031
German	4.73	0.46	4.67	0.46	.85***	0.064***	0.26	0.01	7.37	0.25	0.116	0.090	-0.026
English	4.71	0.61	4.65	0.62	.91***	0.057***	0.26	0.01	6.36	0.22	0.104	0.081	-0.024
French	4.59	0.69	4.54	0.68	.89***	0.051***	0.32	0.01	4.72	0.16	0.125	0.088	-0.037
T2 (Grade 10)													
Mathematics	4.51	0.82	4.44	0.78	.86***	0.070***	0.42	0.02	4.22	0.17	0.172	0.121^bc^	-0.051
German	4.70	0.57	4.66	0.52	.76***	0.036*	0.38	0.02	2.39	0.09	0.147	0.092^b^	-0.056
English	4.71	0.65	4.67	0.62	.86***	0.043**	0.34	0.01	3.24	0.13	0.136	0.090^c^	-0.046
French	4.55	0.73	4.51	0.70	.84***	0.043**	0.41	0.02	2.65	0.10	0.154	0.098	-0.056
T3 (Grade 11)													
Mathematics	4.50	0.80	4.42	0.82	.88***	0.081***^a^	0.39	0.02	5.08	0.21	0.170	0.125	-0.045^d^
German	4.75	0.57	4.72	0.54	.77***	0.030*^a^	0.38	0.02	1.98	0.08	0.186	0.108	-0.078^d^
English	4.72	0.62	4.65	0.62	.81***	0.068***	0.38	0.02	4.39	0.18	0.163	0.115	-0.048
French	4.48	0.73	4.44	0.72	.85***	0.044**	0.40	0.02	2.68	0.11	0.147	0.096	-0.052

*M* = Mean; *SD* = Standard deviation; *SE* = Standard error; *t* = t-value; *d* = Cohen’s *d*; *M*_*sg*_ = Mean self-reported grades; *M*_*ag*_ = Mean actual grades; *M*_*Diff_sg-ag*_ = Mean of self-reported minus actual grades*; M*_|Diff_sg-ag|_ = Mean of absolute misreporting*; M*_*Over*_ = Mean of over-reporting*; M*_*Under*_ = Mean of under-reporting; significantly different means are indicated by the same superscript (i.e., ^a,b,c,d^) and all of the significant differences were found to be significant on the *p* < .05 level. Swiss grades range from 1 (worst grade) to 6 (best grade) with 4 being the threshold for a sufficient grade.

* *p* < .05.

** *p* < .01.

*** *p* < .001

#### Self-reported grades

At each assessment, participants were asked to report their most recent midyear grades in mathematics, German, English, and French classes. Table 1 shows the mean scores and standard deviations of self-reported grades.

### Data analysis

Academic grades can be considered as ordinal scale variables, as the meaning of a difference between a grade of 3.5 and a grade of 4 is not necessarily the same as the meaning of a difference between a grade of 5.5 and a grade of 6, even if the difference is actually the same. This issue is even more apparent if grades are represented by letters instead of numbers. Nevertheless, grades are treated as interval scale variables both in practice (e.g., in schools when computing averages) and in research [22]. In order to examine the difference between treating grades as interval instead of ordinal scale variables, we have compared Pearson and Spearman bivariate correlation coefficients. The mean absolute difference between the two coefficients across school subjects and measurement occasions was found to be as low as .02 with an equally low standard deviation of .02 (min. .01; max. .07). Thus, considering the sensible increase in complexity that would result from treating grades as ordinal variables in the analyses described in the following, grades were treated as interval scale variables, which is in line with other studies and increases the comparability of the present results.

The analysis strategy for the examination of the accuracy of self-reported grades from both a correlational and a discrepancies perspective was as follows. Regarding the extent of missing data, a total of 2.8% to 3.3% of the data in the self-reported grades, and 3.7% to 4.4% of the data in the actual grades, was missing across academic subjects at T1 when considering only participants who completed the T1 assessment. The percentages of missing data were found to be 8.3% to 8.5% for self-reported grades and 1.9% to 2.8% for actual grades at T2. At T3, 4.0% to 5.3% of the self-reported grades and 1.5% to 3.1% of the actual grades were missing. Moreover, the pattern of longitudinal missing data was examined using a method developed by Hedeker and Gibbons [23] that compared the latent growth of actual and self-reported achievement of students having compete data to the latent growth for the entire sample in which Full-Information Maximum Likelihood (FIML) was employed to impute missing values. The resulting mean scores were highly similar for each analysis, suggesting that systematic drop-out was not present in this study. The overall situation regarding missing data in self-reported and actual grades was evaluated as unproblematic and no further steps were undertaken to address missing data in present analyses for the following reasons: First, percentages of missing data below 5% are largely not considered problematic [24] and percentages below 10% are unlikely to lead to biased estimates [25]. Second, the sample size of the present study was large thereby precluding an appreciable loss of power due to missing data. Third, longitudinal attrition was not a factor (see above). Lastly, the default option of Full-Information Maximum Likelihood (FIML) is not available in models with exclusively manifest variables, such as the models presented below.

As for the correlational perspective, correlations between self-reported and actual grades were computed. Regression analyses were subsequently used to examine the reliability of self-reported grades across the four academic subjects, with moderation analyses conducted using M*plus* 7.11 [26]. Academic subject was modeled as a dummy-coded moderator of the effect of self-reported grades on actual grades, with self-reported grades grand mean centered separately for each subject. Gender and age were included as covariates and clustering within classes was taken into account using the sandwich estimator.

The second step employed the discrepancies perspective involving self-report/actual grade discrepancy variables. First, the difference between self-reported and actual grades was computed (i.e., Diff___sg-ag) with the mean of these scores representing mean misreporting (*M*_Diff_sg-ag_). We also calculated mean absolute misreporting (*M*_|Diff_sg-ag|_) by computing the mean of the absolute values of the differences between self-reported and actual grades. Over- and under-reporting variables were then computed. Over-reporting was calculated by recoding negative values to 0, with the mean score of this variable (*M*_*Over*_) representing mean over-reporting. To calculate under-reporting, positive values were recoded as 0, with the mean score of this variable (*M*_*Under*_) indicating mean under-reporting. These variables were created separately for each academic subject and assessment phase.

Once all relevant variables were calculated, a series of analyses was performed to examine if the mean misreporting *M*_Diff_sg-ag_ between self-reported and actual grades was significant and if there were differences in mean misreporting *M*_Diff_sg-ag_, and absolute misreporting *M*_|Diff_sg-ag|_ among the four academic subjects. We further analyzed the amount of over-reporting (*M*_*Over*_) and under-reporting (*M*_*Under*_) as well as whether they differed across academic subjects. To examine mean misreporting *M*_Diff_sg-ag_, we used paired sample *t*-tests. Differences among academic subjects in mean misreporting *M*_Diff_sg-ag_, absolute misreporting *M*_|Diff_sg-ag|_, as well as over-reporting (*M*_*Over*_) and under-reporting (*M*_*Under*_), were examined using regression analyses in M*plus* with dummy coded subject area variables (mathematics as reference category). For each of the four dependent variables, all analyses were conducted separately for each of the three measurement occasions to examine consistency of results.

## Results

### Correlational perspective

The correlations between self-reported and actual academic grades are reported in Table 1 and varied between .76 and .93, with an average Fisher-Z standardized and re-standardized correlation of .85 [27]. All correlations were highly significant, highlighting the consistent reliability of self-reported grades. Results from the moderation analyses to examine the reliability of self-reported grades are reported in Table 2.

**Table 2:** Summary of moderation analyses for variables predicting actual grades (N_T1_ = 3431; N_T2_ = 2427; N_T3_ = 2174).

	T1 (Grade 9)			T2 (Grade 10)			T3 (Grade 11)		
	*B*	*SE B*	*β*	*B*	*SE B*	*β*	*B*	*SE B*	*β*
Intercept	4.54	0.02		4.47	0.02		4.44	0.02	
Self-reported grade	0.95	0.02	0.93***	0.82	0.05	0.86***	0.92	0.04	0.90***
German	0.15	0.02	0.10***	0.23	0.02	0.15***	0.31	0.02	0.19***
English	0.13	0.02	0.09***	0.23	0.03	0.15***	0.22	0.03	0.14***
French	0.02	0.02	0.01	0.09	0.03	0.06**	0.05	0.02	0.03*
Self-reported grade*German	-0.10	0.03	-0.04**	-0.16	0.07	-0.07*	-0.11	0.05	-0.04*
Self-reported grade*English	-0.02	0.03	-0.01	-0.01	0.06	0.00	-0.05	0.04	-0.02
Self-reported grade*French	-0.05	0.03	-0.03	-0.04	0.03	-0.02	-0.11	0.05	-0.06*
Sex (male)	-0.05	0.01	-0.04**	-0.07	0.03	-0.05**	-0.05	0.02	-0.04**
Age	-0.02	0.01	-0.02**	-0.01	0.02	-0.01	-0.04	0.02	-0.03

Note. *R*^2^_T1_ = .83; *R*^2^_T2_ = .71; *R*^2^_T3_ = .75; Mathematics was used as a reference category for the dummy-coding of academic subjects.

* *p* < .05.

** *p* < .01.

*** *p* < .001.

In the first assessment at grade 9, self-reported grades for German were found to have slightly lower reliability than for mathematics. At the grade 10 assessment, self-reported grades for German were again found to be slightly less reliable than for mathematics. In grade 11, self-reported grades in German remained slightly less reliable than for mathematics, with self-reported grades in French additionally found to have lower reliability than mathematics. Concerning the covariate measures, males had lower actual grades in mathematics than females at each assessment, with older students obtaining lower actual grades in mathematics in the grade 9 assessment.

### Discrepancies perspective

**Fig 1:**
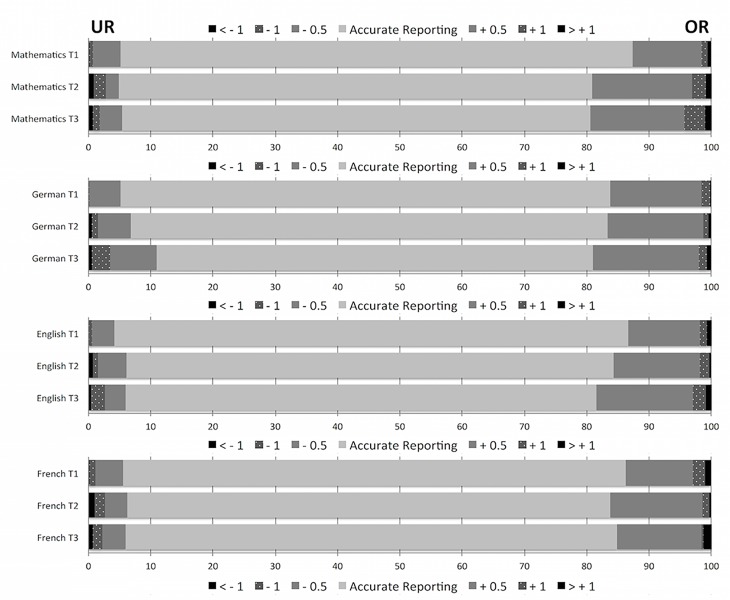
Cumulative percentages of mean differences between self-reported and actual grades. UR = Under-reporting; OR = Over-reporting.

The mean misreporting *M*_Diff_sg-ag_ and paired sample *t*-test results for all four academic subjects and each assessment phase are reported in Table 1. On average, students significantly over-reported their actual grades in all academic subjects from grades 9 through 11. However, the effect sizes for over-reporting were small, with Cohen’s *d* ranging from 0.08 to 0.25 (median *d* = 0.16). Fig 1 illustrates the cumulative percentages of mean differences between self-reported and actual grades across grades 9 to 11 (0 = accurate reporting, negative = under-reporting, positive = over-reporting). The percentage of students under-estimating their grade was ~5% and relatively constant over time, with the single exception of German in grade 11 (~10%). In contrast, the percentage of students over-estimating their grade was ~15% in grade 9 and increased slightly to ~20% by grade 11. In other words, the percentage of students found to accurately report their grade was just over 80%, with students becoming slightly less accurate over time in all academic subjects.

Cumulative percentages of different levels of mean differences between self-reported and actual grades (computed by subtracting the actual grade from the self-reported grade) at the three assessment phases for each subject domain. This scaling is based on the Swiss grading system which ranges from 1 (poorest grade) to 6 (best grade) with 4 being the threshold for a passing grade. Levels of under-reporting of more than one grade are displayed as a single level of under-reporting (labeled as “< (-1)”). The same is true for over-reporting (labeled “> (1)”). Negative and positive deviations from 0 of the same absolute magnitude are displayed in the same color/pattern.

As for differences in mean misreporting (*M*_Diff_sg-ag_) with respect to mathematics vs. verbal domains, results showed no significant differences across assessments with one exception: German had a lower mean difference than mathematics in grade 11 (see Table 1; *p* < .05). Absolute misreporting *M*_|Diff_sg-ag|_ is also outlined in Table 1 for each subject and assessment phase. *M*_|Diff_sg-ag|_ values were notably low and ranged from 0.10 to 0.19 (median: 0.15), with analyses of differences in absolute misreporting across subjects yielding no significant differences at any assessment phase. Over-reporting (*M*_*Over*_) values ranged from 0.08 to 0.13 (median: 0.09) and under-reporting (*M*_*Under*_) values ranged from -0.02 to -0.08 (median: -0.05) across subjects and assessment phases (see Table 1). Results showed few differences in over- and under-reporting between the mathematics and verbal domains: over-reporting was higher for mathematics than in German and English in grade 10 (*p* < .05), and under-reporting was higher for German than mathematics in grade 11 (*p* < .05).

## Discussion

The present study examined the accuracy of self-reported academic grades in mathematics, German, English, and French in a sample of Swiss high-school students from grades 9 through 11. Our analytical approach adopted two perspectives, namely a *correlational perspective* in which bivariate relations between self-reported and actual grades were examined (i.e., reliability of self-reported grades as indicators of actual grades) and a *discrepancies perspective* in which differences in the observed levels of self-reported and actual grades were examined.

### Correlational perspective

The correlation between self-reported and actual grades was significant, positive, and substantial in all academic subjects across grades 9 to 11 showing self-reported grades to be reliable indicators of actual grades. These results are in line with our hypotheses and both replicate and extend previous results on the reliability of self-reported grades [1,8]. However, after controlling for gender and age, the reliability of self-reported grades was not equivalent across academic subjects, with results showing self-reported grades in German to be less reliable than for mathematics. Although the respective effect sizes were found to be small, they were nonetheless consistently observed over a two-year period.

Previous research suggests that homogeneity of subject area content and perceived arbitrariness of grading might be responsible for these subject area effects. As for content homogeneity, Goetz et al. [28] on academic self-concept and emotions noted that the content of German class (i.e., native language of most study participants) is characteristically broad in scope, particularly in comparison to more homogenous mathematics topics. Given that observed relations between academic self-report variables are also weaker in more heterogeneous language domains, it is possible that self-reported grades in language domains may also be less reliable due to content heterogeneity. With respect to perceived arbitrariness of grading practices, objective criteria are prototypically used to define correctness in mathematics. In contrast, more ambiguous criteria are employed in social science classes (e.g., languages). Thus, actual grades might be perceived as less arbitrary by students in mathematics as compared to other domains [29]. As such, it is possible that students may also be more prone to over- or under-report their grades in academic subjects that are more arbitrary in terms of grading (e.g., native language classes).

The reliability levels of self-reported grades in second language classes (i.e., English and French) were not found to be lower than for mathematics, with the exception of the reliability of French self-reported grades in grade 11. Although French does represent one of four national languages in Switzerland, it is nonetheless considered a foreign language for most study participants, similar to English. Accordingly, it is possible that given the necessarily more restricted nature of second-language (i.e., basic grammar and reading of simple texts) relative to first-language content (i.e., studies of highly complex literature), English and French were likely more similar to mathematics with respect to content homogeneity than was German, despite similar grading practices. Nevertheless, apart from subject domain differences due to first-language content, findings from a correlational perspective suggest that self-reported grades are generally accurate and reliable indicators of actual grades across domains from grades 9 to 11.

### Discrepancies perspective

Similar to previous research [1,8], our study findings also demonstrated a consistent tendency for students to over-report their grades over time and across academic subjects. Although in line with our study hypotheses, it should be noted that the effect sizes for over-reporting were small with the median Cohen’s *d* across domains and assessment being 0.16. These results suggest that observed differences between self-reported and actual grades do not simply reflect biased recall or random error, but also a systematic tendency to portray one’s academic performance as slightly better than it actually is. This tendency has been extensively examined as a self-enhancement or impression management strategy [11,30–32] that was found to corresponds to long-term deficits despite short-term benefits: Whereas this strategy may prove effective for enhancing positive mood, self-concept, and self-esteem, it nonetheless may also put students at risk for developing maladaptive learning strategies and poor performance [31,33].

Our examination of differences in mean misreporting also revealed no significant differences between academic subjects in grades 9 and 10, with the only difference found in grade 11 with German classes corresponding to lower mean misreporting levels than mathematics classes. More specifically, this finding indicates when students reached grade 11, the ratio of under-reporting to over-reporting found concerning their grades in German was more balanced than for mathematics, with the overall random error variance thus being higher in German as compared to mathematics classes. However, as this result was found at only one assessment phase, it warrants replication in future studies. As for absolute misreporting, we found that a median value of 0.15 (range: [0.10; 0.19]) which is rather low given the 6-point Swiss grading system (1 = poorest grade, 6 = best grade). Moreover, differences in absolute misreporting, over-reporting, or under-reporting with respect to language versus mathematics domains were generally not significant at any assessment phase, with scattered differences being notably small in magnitude. In sum, our discrepancies perspective shows self-reported grades be considerably accurate in differing only slightly from actual grades, despite a small yet systematic tendency for over-reporting.

### Self-reported vs. actual grades: What to use?

Our study findings showed the accuracy of self-reported grades to be high from both a correlational and a discrepancies perspective. Considering that self-reported grades are usually more accessible than students’ actual grades, it could be argued that self-reported grades are to be preferred over actual grades for the sake of research efficiency. However, Kuncel et al. [1] note that the choice between self-reported and actual grades should not solely be based on practical expediency but rather the research question at hand. For example, whereas research on how specific grading practices impact student persistence warrant objective grade measures, self-reported grades might be more useful if the aim is to instead directly explore the association between students’ perceived course performance and overlapping constructs such as subject-matter interest or academic emotions.

## Strengths and limitations

To the best of our knowledge, this study is the first to demonstrate the accuracy of self-reported grades as indicators of actual grades in the Swiss school system both from a correlational and from a discrepancies perspective, with observed discrepancies being notably small across four academic subjects and grades 9 to 11. However, as the present sample was composed entirely of high-school students, it is possible that cognitive ability levels may have contributed to greater accuracy thus preventing our findings from generalizing to elementary- or middle-school students. Additionally, another limitation is that the validity was examined only for a single non-language domain (mathematics). Accordingly, further study with younger samples across a greater variety of both natural and social science domains is needed. An examination of the developmental pattern of the accuracy of self-reported grades in different academic subjects might also be examined in future research in which a more extensive time frame across childhood and adolescence is assessed. In this regard, Schneider and Sparfeldt [10] reported that correlations between self-reported and actual grades increased markedly from the 2^nd^ through the 4^th^ class level and had reached a level that was comparable to other studies. Although the longitudinal development of the correlation between self-reported and actual grades was not the focus of the present study, the results presented in Table 1 suggest that although correlations dropped from T1 to T2, they nonetheless remained high and were stable from T2 to T3 for all academic subjects.

## Conclusion

The present study replicated and extended results from previous research in showing that self-reported academic grades are accurate indicators of actual grades. Self-reported grades were found to correlate strongly with actual grades and did not differ substantially in terms of specific discrepancy levels. We were further able to replicate that students do, on average, consistently tend to over-report their grades. Our correlational and discrepancy findings were rather consistent across grade levels and academic domains. Although our findings suggest that self-reported grades in native language classes may be slightly less reliable than in other subjects, we can nonetheless conclude that self-reported grades may be generally used as accurate indicators of actual grades in educational studies.

## Supporting information

S1 Data Data used in the present study. (SAV)
